# Efficacy of a cognitive-behavioral digital therapeutic on psychosocial outcomes in rheumatoid arthritis: randomized controlled trial

**DOI:** 10.1038/s44184-024-00085-8

**Published:** 2024-09-03

**Authors:** Linda T. Betz, Gitta A. Jacob, Johannes Knitza, Michaela Koehm, Frank Behrens

**Affiliations:** 1 Research Department, GAIA, Hamburg, Germany; 2 Institute for Digital Medicine, University Hospital of Giessen and Marburg, Philipps University Marburg, Marburg, Germany; 3 Fraunhofer Institute for Translational Medicine and Pharmacology ITMP, Frankfurt am Main, Germany; 4 Fraunhofer Cluster of Excellence Immune Mediated Diseases CIMD, Frankfurt am Main, Germany; 5 Department of Rheumatology, Goethe University Frankfurt, University Hospital, Frankfurt am Main, Germany

**Keywords:** Outcomes research, Rheumatoid arthritis, Patient education, Quality of life

## Abstract

Cognitive behavioral therapy improves psychosocial outcomes in rheumatoid arthritis (RA), but access is limited. We conducted a randomized controlled trial to evaluate the efficacy of a cognitive-behavioral digital therapeutic, *reclarit*, on psychosocial outcomes in adult RA patients with impaired health-related quality of life. Participants were randomized to *reclarit* plus treatment as usual (TAU) or TAU plus educational and informational material (active control). The primary outcome was SF-36 mental (MCS) and physical (PCS) component summary scores at 3 months, with additional assessments at 6 months. *reclarit* significantly improved SF-36 MCS scores compared to control (mean difference 3.3 [95% CI 0.7, 5.9]; p = 0.014), with high user satisfaction and sustained improvements at 6 months. Depression, anxiety, fatigue, and social/work functioning also improved significantly, while SF-36 PCS, pain, and disability scores did not differ. In conclusion, *reclarit* offers immediate, effective, evidence-based and personalized psychological support for RA patients.

## Introduction

Rheumatoid arthritis (RA) is a chronic inflammatory disorder that affects ~0.6% of individuals worldwide^1^ and up to 1.6% of individuals in Germany^2^. Patients typically initially experience swelling and pain in their joints, which may be followed by joint damage, bone destruction, and severe functional limitations^3^. In addition to impacting physical function, psychosocial distress is common in RA. Depending on the definition and instrument used^4^, ~25–40% of patients experience depression^4,5^ and up to 77% experience anxiety^6^; many patients experience both^7^. Notably, some symptoms, such as fatigue, are linked to RA disease activity^8^ and may be misconstrued as psychosocial distress. However, even with effective control of disease activity through disease-modifying antirheumatic drugs (DMARDs), including biologics and targeted agents, psychosocial outcomes often remain insufficiently improved^9^. This disparity is relevant because psychosocial distress in RA has been shown to be a predictor of increased disease activity, reduced quality of life (QoL), work impairment, and higher mortality^10–14^. Therefore, a holistic approach to RA care is needed, with targeted mental health support for symptoms that may not improve with controlling disease activity^10^.

Accordingly, in addition to pharmacologic therapy, guidelines from the European Alliance of Associations for Rheumatology (EULAR)^15^, the American College of Rheumatology (ACR)^16^, and the German Society for Rheumatology^17^ support multidisciplinary treatment approaches including nonpharmacologic strategies to optimize outcomes in patients with RA. In particular, cognitive behavioral therapy (CBT) has shown positive effects on depression, anxiety, and fatigue in patients with RA^18–21^ and is recommended by the German national guidelines for early RA^17^ and the EULAR guidelines for self-management strategies in patients with inflammatory arthritis^22^.

Despite the proven effectiveness of CBT, access to CBT-based interventions for RA remains limited, and patients with RA continue to report a high disease burden^23^. Recent surveys have demonstrated unmet needs in this patient population as well as limited ability to self-manage their rheumatic conditions^24,25^, especially in the presence of clinically relevant levels of depression and anxiety^26^. Access to treatment is additionally limited by the increasing shortage of specialists^27^. Digital therapeutics are increasingly used to overcome the escalating challenges of traditional care^28^. EULAR guidelines explicitly ask rheumatologists to consider “telehealth for non-pharmacological interventions including but not limited to disease education, advice on physical exercise, self-management strategies and psychological intervention”^29^ and even refer to digital health as “essential” to supporting self-management of inflammatory arthritis^22^.

In December 2019, Germany enacted the Digital Healthcare Act (*Digitale-Versorgung-Gesetz*) that allows provision and reimbursement of digital therapeutics for individuals covered by statutory health insurance^30,31^. Digital therapeutics that meet requirements for reimbursement can be “prescribed” by healthcare providers in the same way as a medication. A national survey found that most patients and rheumatologists agreed that digital tools, including digital therapeutics, could be useful in the management of rheumatic and musculoskeletal disorders, and identified the lack of information and evidence available regarding digital tools as the main barriers^32^. Currently there are no digital therapeutics for RA approved for use in Germany^33^.

**Fig. 1:**
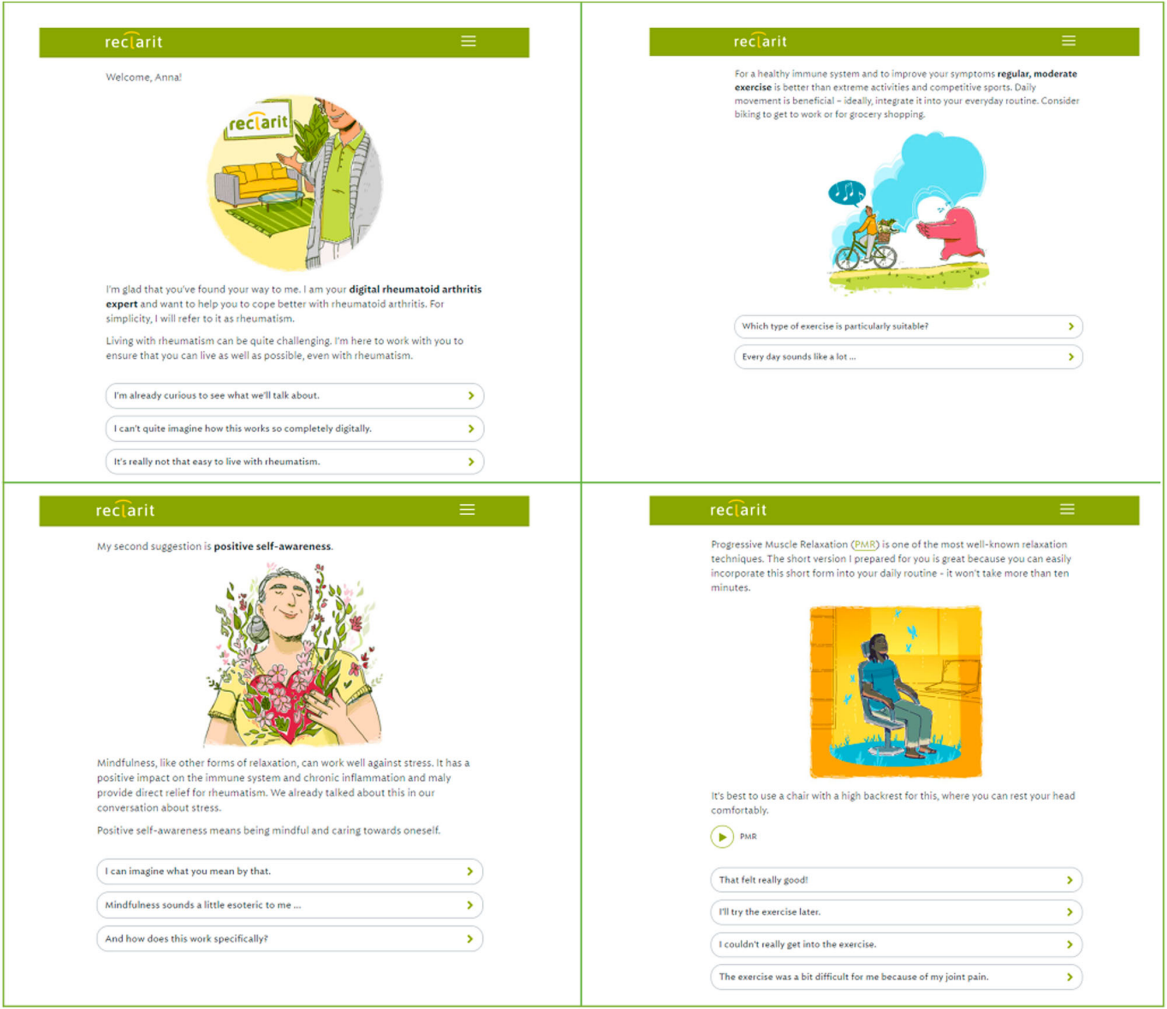
Selected screenshots of *reclarit* converted to English translations for illustrative purposes. The *reclarit* digital therapeutic involved in this study was used exclusively in the German version. An English version is not currently available.

We developed a fully automated digital therapeutic called *reclarit* that uses CBT methods and lifestyle counseling to educate RA patients and help them cope psychologically with impairments associated with RA (Fig. 1). This digital therapeutic aims to facilitate improvements in psychosocial outcomes, including QoL, by addressing a broad range of relevant domains, such as coping with stress and pain, reducing fatigue, improving sleep, healthy dieting, physical activity and exercising, smoking cessation, and enhancing social support and self-esteem (Table 1). The aim of this randomized clinical trial (German Clinical Trials Register DRKS00025256) was to assess the efficacy of *reclarit* in patients with RA facing psychosocial distress by comparing psychosocial outcomes in patients receiving *reclarit* with those in patients receiving treatment as usual (TAU) plus educational material (active control group).

**Table 1:** Content of the *reclarit* digital therapeutic

Domain	Topics
Basic education about chronic pain	• Pain and the role of the autonomic nervous system • The parasympathetic nervous system and pain management • Ways of training the parasympathetic system (e.g., breathing exercises)
Interaction between pain and stress	• Role of stress in the parasympathetic system • Interaction between stress and chronic inflammation • Strategies to reduce stress in everyday life
Pain coping	• Pain and counterintuitive actions • Role of active coping • Psychological methods to implement active coping strategies with a focus on exercise and physical activity
Healthy sleep	• Relationship between sleep, fatigue, and chronic inflammation • Sleep hygiene • Changing negative thoughts about sleep • Techniques to improve sleep quality
Healthy lifestyle and diet	• Healthy lifestyle in RA, with a focus on healthy eating • CBT techniques to facilitate the implementation of a healthy diet in everyday life
Mental well-being	• Techniques to reduce stress and improve mental well-being and positive emotional experiences

*CBT* cognitive behavioral therapy, *RA* rheumatoid arthritis.

## Results

### Patient disposition and characteristics

**Fig. 2:**
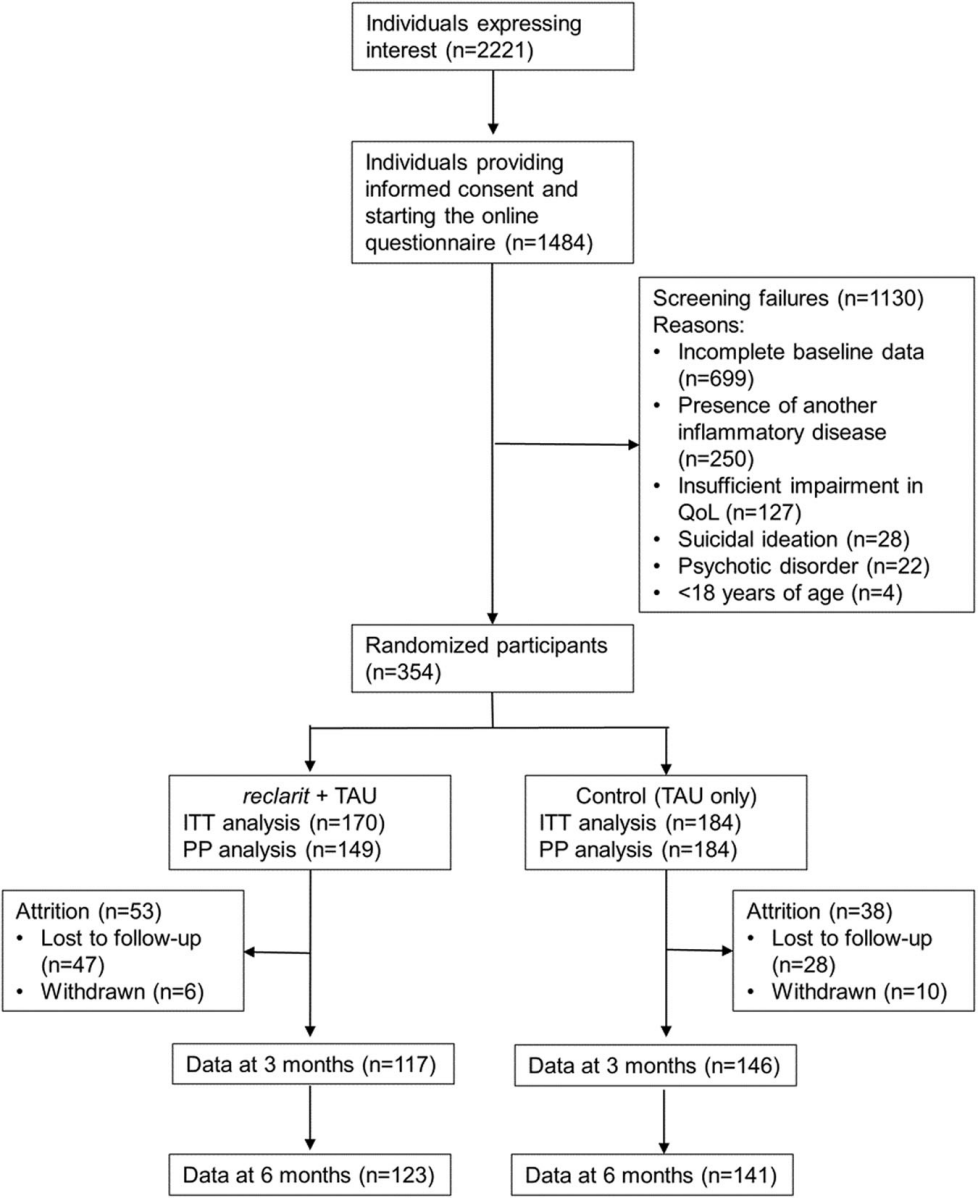
Participant disposition. ITT intention-to-treat, PP per protocol, QoL quality of life, TAU treatment as usual.

A total of 2221 individuals expressed interest in study participation and 1484 provided informed consent and began the online screening questionnaire; of these, 1130 individuals were excluded, primarily due to incomplete baseline data (n = 699) (Fig. 2). A total of 354 individuals met all inclusion criteria and were randomized to *reclarit* (n = 170) or control (n = 184). These groups provided data for the primary analyses based on the intention-to-treat population. At month 3, 31% of the *reclarit* group and 21% of the control group had been lost to follow-up or discontinued the study. Both groups remained generally stable in size between months 3 and 6 (Fig. 2). Almost 90% of patients in the *reclarit* group (149/170; 87.6%) completed at least 2 modules and were therefore included in pre-specified per protocol (PP) analyses. All control group participants were included in PP analyses.

Approximately 90% of participants in this study were self-reported females (312/354 [88.1%]). Participants had experienced RA symptoms for a mean of more than 9 years and had received a diagnosis of RA ~7 years previously, on average (Table 2). Anti-rheumatic therapy was being used by 85.9% (304/354), most commonly a conventional synthetic DMARD (csDMARD). csDMARD and biologic DMARD (bDMARD) use was numerically lower in the *reclarit* group than in the control group (43.5% vs 51.6% for csDMARDs; 20.6% vs 24.5% for bDMARDs). Psychotherapy use did not differ between the groups (Table 2). Over the study period, differences between the two groups in the percentages of participants receiving specific treatments were not statistically significant (Table 3).

**Table 2:** Baseline demographic and clinical characteristics

Characteristic	*reclarit*	Control	Total
n	170	184	354
Female, n (%)	150 (88.2%)	162 (88.0%)	312 (88.1%)
Age, mean (SD)	50.9 (11.8)	50.0 (14.7)	50.4 (13.4)
Diagnosis, n (%)			
Seropositive RA^a^	39 (22.9%)	40 (21.7%)	79 (22.3%)
Seronegative RA^b^	28 (16.5%)	29 (15.8%)	57 (15.2%)
Unspecified RA^c^	103 (60.6%)	115 (62.5%)	218 (61.6%)
Years since onset of RA symptoms, mean (SD)	10.4 (9.4) [n = 48]	8.7 (9.1) [n = 78]	9.3 (9.2) [n = 126]
Years since first diagnosis of RA, mean (SD)	7.4 (7.8) [n = 55]	6.5 (8.5) [n = 84]	6.8 (8.2) [n = 139]
Living situation, n (%)			
Alone	43 (25.3%)	44 (23.9%)	87 (24.6%)
Alone with children	7 (4.1%)	13 (7.1%)	20 (5.6%)
With partner without children	63 (37.1%)	69 (37.5%)	132 (37.3%)
With partner with children	51 (30.0%)	50 (27.2%)	101 (28.5%)
Shared apartment	6 (3.5%)	8 (4.3%)	14 (4.0%)
DMARD or analgesic treatment, n (%)			
Any anti-rheumatic treatment (multiple answers possible)	146 (85.9%)	158 (85.9%)	304 (85.9%)
csDMARDs	74 (43.5%)	95 (51.6%)	169 (47.7%)
bDMARDs	35 (20.6%)	45 (24.5%)	80 (22.6%)
TNF inhibitors	23 (13.5%)	28 (15.2%)	51 (14.4%)
Other bDMARDs	12 (7.1%)	17 (9.2%)	29 (8.2%)
JAK inhibitors	10 (5.9%)	19 (10.3%)	29 (8.2%)
Glucocorticoids	61 (35.9%)	81 (44.0%)	142 (40.1%)
NSAIDs	72 (42.4%)	64 (34.8%)	136 (38.4%)
Opioids	8 (4.7%)	14 (7.6%)	22 (6.2%)
Other	5 (2.9%)	1 (0.5%)	6 (1.7%)
Currently in physiotherapy, n (%)	44 (25.9%)	51 (27.7%)	95 (26.8%)
Currently in psychotherapy, n (%)	21 (12.0%)	23 (16.0%)	44 (14.0%)

Data are presented as mean (standard deviation) or n (%). Percentages may not equal 100 due to rounding.

*bDMARD* biologic DMARD, *csDMARD* conventional synthetic DMARD, *DMARD* disease-modifying antirheumatic drug, *JAK* Janus kinase, *NSAID* nonsteroidal anti-inflammatory drug, *TNF* tumor necrosis factor, *RA* rheumatoid arthritis, *SD* standard deviation.

^a^ICD-10 codes M0.5.0, M0.5.8, and M0.5.9.

^b^ICD-10 codes M06.0 and M06.8.

^c^ICD-10 code M06.9 or diagnosis of RA with no information regarding the location or rheumatoid factor status.

**Table 3:** Treatment characteristics during the study period

Treatment	3 months			6 months		
	*reclarit*	Control	p value	*reclarit*	Control	p value
n	117	146	NA	123	141	NA
DMARD or analgesic treatment, n (%)						
Any anti-rheumatic medication (multiple answers possible)	98 (83.8%)	119 (81.5%)	0.633	105 (85.4%)	121 (85.8%)	0.917
csDMARDs	49 (41.9%)	73 (50.0%)	0.190	52 (42.3%)	68 (48.2%)	0.333
bDMARDs	29 (24.8%)	39 (26.7%)	0.723	33 (26.8%)	36 (25.5%)	0.811
TNF inhibitors	21 (17.9%)	24 (16.4%)	0.747	24 (19.5%)	22 (15.6%)	0.404
Other bDMARDs	8 (6.8%)	15 (10.3%)	0.447	9 (7.3%)	14 (9.9%)	0.595
JAK inhibitors	6 (4.3%)	16 (11.0%)	0.079	10 (8.1%)	15 (10.6%)	0.629
Glucocorticoids	40 (34.2%)	47 (32.2%)	0.732	38 (30.9%)	39 (27.7%)	0.564
NSAIDs	48 (41.0%)	52 (35.6%)	0.369	49 (39.8%)	45 (31.9%)	0.180
Opioids	6 (5.1%)	7 (4.8%)	1.00	4 (3.3%)	7 (5.0%)	0.700
Other	4 (3.4%)	3 (2.1%)	0.766	8 (6.5%)	6 (4.3%)	0.591
Currently in physiotherapy, n (%)	33 (28.2%)	54 (37.0%)	0.127	42 (34.1%)	52 (36.9%)	0.644
Currently in psychotherapy, n (%)	14 (12.0%)	23 (15.8%)	0.478	14 (11.4%)	21 (14.9%)	0.401

P values were determined by chi-square tests.

*bDMARD* biologic DMARD, *csDMARD* conventional synthetic DMARD, *DMARD* disease-modifying antirheumatic drug, *JAK* Janus kinase, *NA* not applicable, *NSAID* nonsteroidal anti-inflammatory drug, *TNF* tumor necrosis factor.

### SF-36 outcomes

The primary endpoint was health-related QoL, as assessed by the Short Form-36 (SF-36; RAND version of the Medical Outcomes Study)^34^, at 3 months in the ITT population. SF-36 mental component summary (MCS) scores in the *reclarit* group were statistically significantly superior to the control group in the ITT population at 3 months (Table 4). The treatment effect was 3.3 (95% CI 0.7, 5.9) and Cohen’s *d* effect size was 0.23 in favor of *reclarit*. Similar results were observed in the ITT population at 6 months and in the PP population at 3 and 6 months. SF-36 physical component summary (PCS) scores were not significantly different between the *reclarit* and control groups in the ITT and PP populations at either time point (Table 4). In post-hoc analyses of responder rates, SF-36 MCS responder rates in the *reclarit* group were significantly higher than in the control group [36.7% vs 27.5%; p = 0.019].

**Table 4:** SF-36 MCS and PCS outcomes

Outcome and population	Time point	*reclarit*^a^	Control^b^	ANCOVA		Cohen’s *d* (95% CI)^c^
		Mean (SD)	Mean (SD)	Treatment effect (95% CI)	p value	
SF-36 MCS						
ITT	Baseline	33.6 (9.8)	34.2 (9.7)	–		
	3 months	38.6 (12.8)	35.7 (12.6)	3.3 (0.7, 5.9)	0.014	0.23 (0.001, 0.46)
	6 months	39.4 (12.6)	36.4 (13.5)	3.3 (0.5, 6.2)	0.020	0.23 (−0.007, 0.46)
PP	Baseline	34.2 (9.7)	34.2 (9.7)	–		
	3 months	39.3 (12.7)	35.7 (12.6)	3.6 (0.8, 6.5)	0.013	0.29 (0.04, 0.54)
	6 months	39.8 (12.5)	36.5 (13.5)	3.3 (0.4, 6.3)	0.025	0.26 (0.01, 0.50)
SF-36 PCS						
ITT	Baseline	32.8 (8.2)	33.7 (8.6)	–		
	3 months	37.7 (10.7)	37.2 (10.6)	1.1 (−0.9, 3.1)	0.269	0.04 (−0.18, 0.27)
	6 months	39.0 (11.4)	38.7 (11.6)	0.9 (−1.4, 3.2)	0.419	0.03 (−0.20, 0.25)
PP	Baseline	32.7 (8.2)	33.7 (8.6)	–		
	3 months	37.9 (10.7)	37.2 (10.6)	1.4 (−0.7, 3.5)	0.205	0.06 (−0.17, 0.30)
	6 months	39.0 (11.3)	38.7 (11.6)	1.0 (−1.4, 3.5)	0.417	0.03 (−0.22, 0.27)

*ANCOVA* analysis of covariance, *CI* confidence interval, *ITT* intention-to treat, *MCS* mental component summary, *PCS* physical component summary, *PP* per protocol, *SD* standard deviation, *SF-36* Short Form-36.

The pre-specified primary outcome was SF-36 MCS and PCS scores in the ITT population at 3 months

^a^n = 170 for ITT population and 149 for PP population.

^b^n = 184 for ITT and PP populations.

^c^Based on observed values; positive values indicate effects in favor of the *reclarit* group.

### Secondary and post-hoc outcomes

Significant treatment effects in the *reclarit* group vs the control group were observed at 3 months for depression, anxiety, fatigue, and social and work-related functioning in both the ITT and PP populations (Table 5). These treatment effects were maintained at 6 months, except for anxiety (p = 0.051). Differences between groups in pain and Health Assessment Questionnaire-Disability Index (HAQ-DI) were not significant (Table 5).

**Table 5:** Secondary outcomes

Outcome and population	Time point	*reclarit*^a^	Control^b^	ANCOVA		Cohen’s *d* (95% CI)^c^
		Mean (SD)	Mean (SD)	Treatment effect (95% CI)	p value	
Depression (PHQ-9)						
ITT	Baseline	10.8 (4.4)	10.4 (4.4)	–		
	3 months	8.8 (4.8)	10.2 (5.0)	−1.7 (−2.6, −0.8)	<0.001	0.30 (0.07, 0.53)
	6 months	8.6 (4.7)	9.4 (5.0)	−1.1 (−2.0, −0.1)	0.027	0.17 (−0.06, 0.40)
PP	Baseline	10.7 (4.4)	10.4 (4.4)	–		
	3 months	8.6 (4.8)	10.2 (4.9)	−1.8 (−2.7, −0.9)	<0.001	0.32 (0.09, 0.56)
	6 months	8.4 (4.7)	9.4 (5.0)	−1.2 (−2.1, −0.2)	0.018	0.20 (−0.04, 0.43)
Anxiety (GAD-7)						
ITT	Baseline	7.5 (4.8)	7.0 (4.2)	–		
	3 months	6.0 (4.4)	7.0 (4.4)	−1.3 (−2.1, −0.4)	0.003	0.23 (−0.01, 0.47)
	6 months	5.8 (4.0)	6.4 (4.3)	−0.8 (−1.7, 0.02)	0.054	0.15 (−0.09, 0.38)
PP	Baseline	7.4 (4.8)	7.0 (4.2)	–		
	3 months	5.8 (4.3)	7.0 (4.4)	−1.4 (−2.3, −0.6)	<0.001	0.28 (0.03, 0.52)
	6 months	5.8 (4.0)	6.4 (4.3)	−0.8 (−1.6, 0)	0.051	0.15 (−0.09, 0.38)
Fatigue (BRAF-MDQ)						
ITT	Baseline	33.8 (12.4)	32.1 (12.5)	–		
	3 months	29.1 (13.5)	31.9 (13.9)	−4.0 (−6.6, −1.4)	0.003	0.21 (−0.02, 0.44)
	6 months	27.7 (13.9)	30.7 (14.5)	−4.2 (−6.9, −1.5)	0.002	0.22 (−0.02, 0.045)
PP	Baseline	33.0 (11.8)	32.1 (12.5)	–		
	3 months	28.1 (13.0)	31.9 (13.9)	−4.5 (−7.1, −1.9)	<0.001	0.29 (0.05, 0.53)
	6 months	27.0 (13.1)	30.6 (14.5)	−4.3 (−7.0, −1.5)	0.002	0.26 (0.02, 0.51)
Social and work-related functioning (WSAS)						
ITT	Baseline	18.5 (8.0)	16.6 (8.4)	–		
	3 months	15.0 (8.6)	15.5 (8.7)	−1.8 (−3.4, −0.2)	0.028	0.06 (−0.19, 0.30)
	6 months	14.2 (8.5)	14.9 (9.1)	−1.9 (−3.6, −0.2)	0.029	0.08 (−0.16, 0.31)
PP	Baseline	18.1 (7.6)	16.6 (8.4)	–		
	3 months	14.6 (8.4)	15.5 (8.7)	−1.9 (−3.5, −0.3)	0.024	0.10 (−0.14, 0.34)
	6 months	13.9 (8.3)	14.9 (9.1)	−1.9 (−3.6, −0.1)	0.036	0.11 (−0.13, 0.35)
Pain NRS						
ITT	Baseline	6.2 (2.0)	5.9 (1.9)	–		
	3 months	4.8 (2.3)	4.7 (2.2)	−0.04 (−0.55, 0.47)	0.886	0.04 (−0.21, 0.28)
	6 months	4.6 (2.2)	4.3 (2.4)	0.2 (−0.3, 0.7)	0.474	0.13 (−0.11, 0.36)
PP	Baseline	6.2 (2.0)	5.9 (1.9)	–		
	3 months	4.7 (2.3)	4.7 (2.2)	−0.1 (−0.6, 0.4)	0.667	0 (−0.24, 0.24)
	6 months	4.5 (2.2)	4.3 (2.4)	0.2 (−0.4, 0.7)	0.564	0.11 (−0.14, 0.36)
Physical function (HAQ-DI)						
ITT	Baseline	1.1 (0.6)	1.0 (0.6)	–		
	3 months	1.0 (0.6)	0.9 (0.6)	−0.05 (−0.14, 0.05)	0.339	0.05 (−0.17, 0.27)
	6 months	0.3 (0.6)	0.9 (0.6)	−0.04 (−0.13, 0.06)	0.440	0.06 (−0.15, 0.28)
PP	Baseline	1.1 (0.6)	1.0 (0.5)	–		
	3 months	0.9 (0.6)	0.9 (0.6)	−0.1 (−0.1, 0)	0.293	0.01 (−0.23, 0.25)
	6 months	0.9 (0.6)	0.9 (0.6)	0 (−0.1, 0.1)	0.520	0.04 (−0.18, 0.27)

*ANCOVA* analysis of covariance, *BRAF-MDQ* Bristol Rheumatoid Arthritis Fatigue-Multi-dimensional Questionnaire, *CI* confidence interval, *GAD-7* 7-item General Anxiety Disorder, *HAQ-DI* Health Assessment Questionnaire-Disability Index, *ITT* intention-to treat, *NRS* numeric rating scale, *PHQ-9* 9-item Patient Health Questionnaire, *PP* per protocol, *SD* standard deviation, *WSAS* Work and Social Adjustment Scale.

^a^n = 170 for ITT population and 149 for PP population.

^b^n = 184 for ITT and PP populations.

^c^Based on observed values; positive values indicate effects in favor of the *reclarit* group.

Post-hoc responder analyses of depression, in which response was defined as a change of 5 points in the Patient Health Questionnaire (PHQ-9) total score in the ITT population, found that the *reclarit* group had approximately twice as many responders as the control group at 3 months (25.6% vs 12.9%; p = 0.002). The *reclarit* group also had more responders at 3 months in analyses of anxiety, defined as a change of 4 points in the 7-item Generalized Anxiety Disorder questionnaire (GAD-7) score in the ITT population (25.3% vs 16.4%; p = 0.048).

### Adverse events and adverse device effects

No adverse events or adverse device effects were observed. As an additional safety analysis, we calculated the percentage of participants who had a lower health-related QoL at 3 months compared with baseline. This analysis was based on participants with complete observations only (n = 117 for the *reclarit* group and n = 146 for the control group). Lower SF-36 MCS scores at 3 months (indicating greater impairment) were reported by 34.1% of *reclarit* group participants and 43.8% of control group participants (p = 0.143). Lower SF-36 PCS scores at 3 months were reported by 24.7% of *reclarit* group participants and 36.3% of control group participants (p = 0.159).

### User satisfaction

User satisfaction with *reclarit*, as assessed by the Net Promoter Score (NPS) at 3 months (n = 116), was +19.8 (54 [47.4%] promoters and 32 [27.6%] detractors), indicating good user satisfaction.

### Subgroup analyses for effect of baseline characteristics on SF-36

**Fig. 3:**
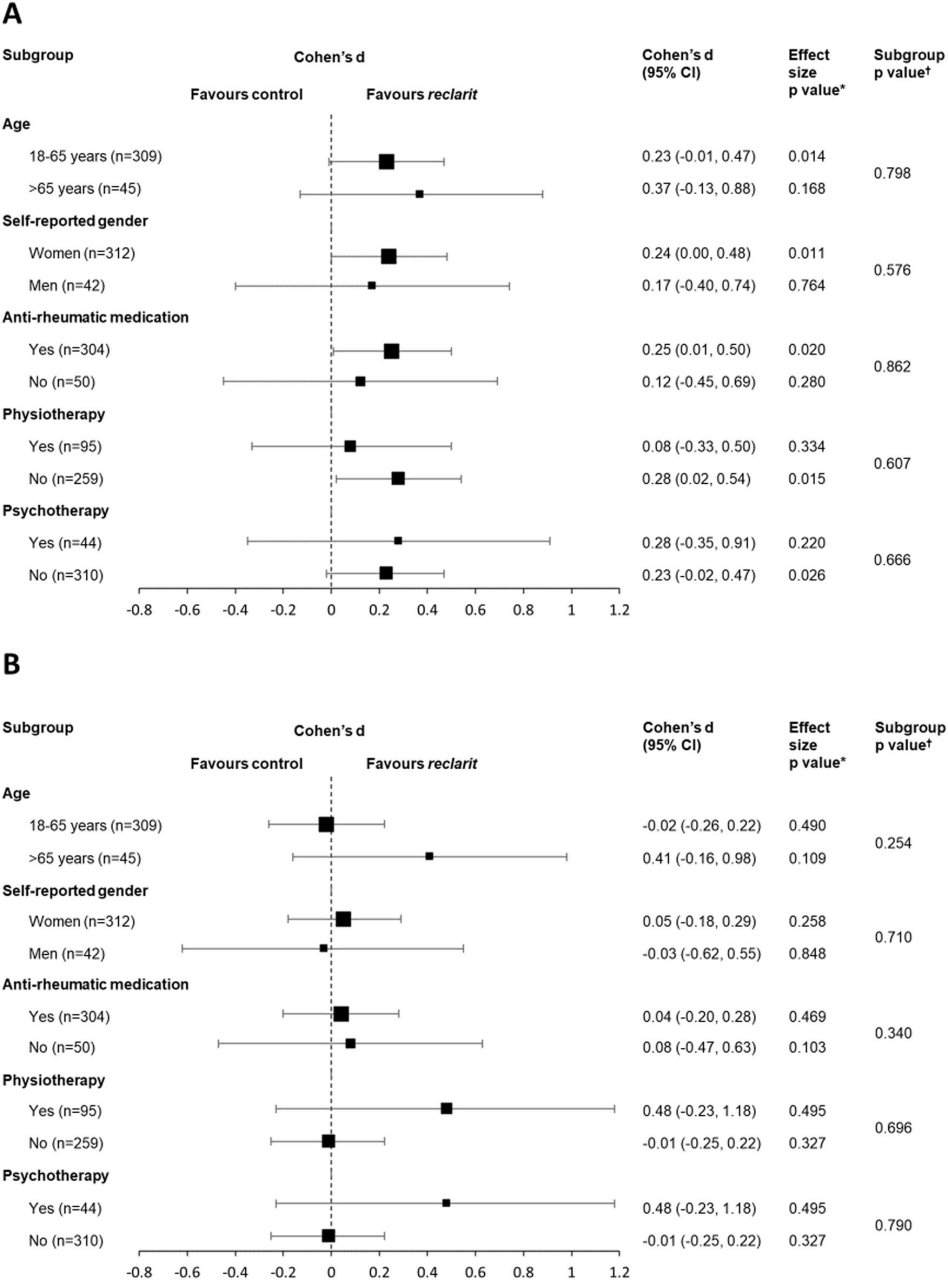
Forest plot of effect sizes (Cohen’s d) at 3 months in the ITT population for. **A** SF-36 MCS and (**B**) SF-36 PCS. Squares indicate Cohen’s d and bars indicate 95% CI.P values are derived from ANCOVA.*P value for the effect size of *reclarit* vs control ^†^P values for subgroup interaction.

We conducted exploratory analyses to gain insight into baseline characteristics that may have affected the *reclarit* treatment response. Analysis of covariance (ANCOVA) analyses did not identify any significant differences in the effect of *reclarit* on SF-36 MCS among subgroups based on age, gender, DMARD use, physiotherapy use, or psychotherapy use (Fig. 3). SF-36 PCS treatment effects were also statistically similar in different subgroups. It is important to note that the study was not powered to analyze statistical differences in subgroups, and statistical analyses may have been impacted by the small number of participants in some subgroups.

## Discussion

This study of 354 participants with RA and impaired health-related QoL demonstrated clinically relevant and sustained improvements in multiple psychosocial outcomes in RA patients using the digital therapeutic *reclarit*. The clinical relevance of these effects was confirmed by responder analyses, which found significantly more responders in the *reclarit* group compared with control (36.7% vs 25.7%). Improvements were also observed in additional psychosocial outcomes, including depression, anxiety, fatigue, and social and work-related functioning. Treatment effects were stable at 6 months and were not specific to age or gender subgroups or to current treatment with DMARDs, physiotherapy, or psychotherapy. Based on these data, we conclude that the fully automated digital therapeutic *reclarit* has the ability to improve psychosocial outcomes in adult patients with RA.

Depression, anxiety, and fatigue are common and debilitating problems in RA that are often refractory to pharmacotherapy^9,35^. The effect sizes at 3 months for reclarit observed in our study for depression (0.30), anxiety (0.23), and fatigue (0.21) are consistent with effect sizes reported in meta-analyses for conventional psychological interventions in RA (depression 0.23^36^, anxiety 0.17^36^, and fatigue 0.32^37^), and the depression effect size exceeds the minimally important difference effect size reported for major depressive disorders (0.24)^38^.

It should be noted that in patients with a disease duration of less than 10 years, the reported effect sizes of psychological interventions on depressive symptoms are ~4-fold larger than in patients with a disease duration of ≥10 years^36^. It is thus possible that prescription of *reclarit* at the time of RA diagnosis might result in an even stronger effect on psychosocial outcomes^39,40^.

As expected, the effects of *reclarit* on psychosocial outcomes were obtained independently of improvement in physical QoL and pain-related outcomes, as no significant between-group differences were observed in these outcomes. These findings are consistent with those from meta-analyses of other psychological interventions in patients with RA^36,41^.

In a recent survey of German patients with RA, patient education and coping were identified as the key unmet needs. Only 25% of surveyed patients described their disease education as “good/very good” and the same proportion were “satisfied/very satisfied” with currently available information. Ninety-one percent rated “coping” as a very important topic, and 48% rated their knowledge in this topic as deficient/insufficient^25^. These data reveal a gap in health services currently provided to RA patients in Germany, and likely elsewhere. Although RA professionals are in strong agreement with the need for holistic RA care, they cite lack of time, training, and staff as key barriers^42^. The decreasing number of rheumatologists in Germany further aggravates existing health service bottlenecks^43^.

Digital therapeutics have the potential to improve access to psychological interventions, including CBT^44^. Patients with RA have shown a high degree of willingness to embrace digital tools^25,28,29^ and promising evidence of the efficacy of these tools is beginning to emerge^45,46^. However, to date no approved digital therapeutics exist for RA patients^33^.

Although digital therapeutics possess significant potential, they may not be universally applicable^47^. Individuals lacking a stable internet connection or those unwilling to engage with such platforms may remain beyond the reach of these programs^48^. Due to the online recruitment method, these individuals were also less likely to be included in the present study. As a result, the present findings may primarily generalize to patients who are both equipped for and motivated to use digital health tools^49^, rather than the broad RA population that might require CBT. Additionally, chronic conditions like RA tend to be more prevalent among older individuals, who may encounter difficulties accessing online offerings^25,48^. In Germany, ~22% of RA patients are 70 or older^2^. Of note, previous analyses of similar digital therapeutics suggest that older participants can engage with and profit from such interventions just as well as their younger counterparts^50^.

As has been found for other internet-based CBT programs^51^, *reclarit* was not associated with detrimental effects on patient outcomes. Participants in this study reported good user satisfaction with *reclarit*; the NPS in this study (+19.8) is in line with other digital applications used for symptom management in RA^28^.

Study limitations include small numbers in some subgroups, including men and patients over 65 years of age. Additionally, for inclusion, patients had to demonstrate a relevant level of impairment in QoL, similar to that of individuals who would most likely be prescribed *reclarit*. Therefore, it remains to be determined how these findings translate to broader contexts, including the important opportunity of preventive use in yet less impaired populations^39,40^.

Dropout rates were consistent with those reported previously for digital applications in RA^52^ but higher in the intervention group compared to the control group. This disparity may be due to participants in the intervention group discontinuing *reclarit* use and study participation upon achieving sufficient benefit, which aligns with the “good enough” effect observed in psychotherapy and digital intervention research^53–55^. In contrast, the control group might have been more motivated to complete follow-ups due to the opportunity to access *reclarit* after 6 months. From a methodological perspective, expected dropout could have been factored into the sample size calculation to ensure sufficient power.

Finally, some outcomes were not prioritized in the present study to minimize participant burden. Specifically, self-efficacy^39,56^ and pain catastrophizing^57^ could offer valuable insights in future research. Pain catastrophizing seems particularly relevant as it could help examine concerns from both healthcare professionals and patients that regular symptom tracking with digital interventions might increase focus on RA symptoms and worsen their impact on daily life^48^. Moreover, given the online setting of the study, physician-derived outcomes such as the Disease Activity Score-28 were not included, and the study did not evaluate the effect of intervention on patients’ global assessment. Studies including this clinical perspective and an economic evaluation are highly warranted.

In conclusion, this randomized controlled trial demonstrated that the digital therapeutic, *reclarit*, offered in addition to TAU, had a significant and clinically relevant beneficial effect on key psychosocial outcomes compared with TAU plus educational material in patients with RA and impaired health-related QoL. These effects were not associated with differences or changes in the medication and other reported treatments of the study participants and can therefore be validly attributed to *reclarit*. Our findings indicate that digital therapeutics such as *reclarit* are well-suited to meeting the needs of RA patients. Thus, complementing pharmacotherapy with the digital therapeutic *reclarit* has the potential to optimize treatment outcomes within a holistic and hybrid framework of RA care^10^. Efforts to implement this digital therapeutic more broadly and evaluate its performance under routine care conditions and in relevant preventive applications^39,40^ therefore appear warranted.

## Methods

### Study design and patients

This prospective, randomized, single-blind study compared the use of *reclarit* plus treatment as usual (TAU) (*reclarit* group) to TAU plus educational material (active control group) in adult patients with RA in Germany (German Clinical Trials Register DRKS00025256). Patients in both groups received medications and other treatments for RA as specified by the treating physician during routine care. Questions and information were provided in German, and all baseline and outcome data were self-reported by participants. The clinical investigation protocol (including an amendment) was reviewed and approved by the ethics committee of the Ärztekammer Hamburg (reference number PV7382). The clinical investigation was conducted in accordance with the ethical principles in the Declaration of Helsinki. Prior to participation, detailed patient information was provided and informed consent was obtained online.

Study participants were recruited via online advertising using Google Ads and through health insurance companies between April 29, 2021 and April 20, 2022. The final data collection date was November 10, 2022. Participants were required to be 18 years of age or older, be diagnosed with RA according to International Classification of Diseases (ICD)-10 (M05-M06) with verification by a doctor’s letter or equivalent proof, be able to access the internet via their own smartphone or computer, have a sufficient understanding of the German language, and provide consent for study participation. Individuals were excluded if they were at risk for suicide, had psychoses, or had non-RA chronic inflammatory diseases as assessed by self-report. An amendment to the trial protocol involving modifications to entry criteria was approved by the ethics committee on April 12, 2022. This amendment required participants to have impaired QoL, defined as SF-36 values of less than 50% of the normal population, in both the MCS and PCS (i.e., SF-36 MCS and SF-36 PCS scores <50). This amendment was made to allow the study to evaluate patients with potential for improvement in QoL.

Participants who met the entry criteria were randomly assigned 1:1 to the *reclarit* or active control group using an online randomization sequence generation software and simple randomization (no blocked randomization or stratification) based on participant identification numbers. Randomization assignments were available to the study manager; this individual was not aware of participant characteristics (ensuring concealed allocation) and was not involved in data collection or analysis. All other study employees were blinded to randomization assignments. Participants were aware of the treatment group to which they had been assigned.

Patient-reported outcome (PRO) data were collected online using a secure, internationally recognized survey software (www.easyfeedback.de). PRO data were recorded at baseline (before initiation of the intervention) and at 3 months and 6 months after initiation of the intervention. Participants were provided with 10€ Amazon vouchers upon completing questionnaires at the 3-month and 6-month follow-ups. Following study completion, individuals in the control group were offered access to *reclarit*.

Pre-specified analyses were conducted on the ITT population (all patients who provided data) and on the PP population (patients who completed at least two modules).

### Interventions

*reclarit* (GAIA, Hamburg, Germany) is a fully automated, internet-based, expert system digital therapeutic for patients with RA, developed in close collaboration with specialists in rheumatology. *reclarit* does not require software installation, runs on the company’s proprietary software, and can be accessed using conventional web browsers on desktop personal computers (PCs), tablet PCs, and smartphones. The digital therapeutic leverages a responsive web design technology with an adaptable website layout that adapts to the size of the display (font size, images) of the accessing device. A group of patients tested a beta version of *reclarit*, and their feedback was integrated to refine the program’s functionality and user experience.

The content in *reclarit* is delivered via text, audio recordings, and images (Fig. 1). Six modules, each of which covers a different domain (Table 1), are organized in the form of individually tailored dialogs in which the user is involved in simulated therapeutic interactions. Users are prompted to react to the information presented by selecting one or several response options. The digital therapeutic then analyses the responses and presents subsequent content according to individual patient needs and therapeutic requirements. Patients are also asked to complete tasks, such as improving dietary habits, and are provided with more specific content (worksheets and summary sheets) as needed. Users can pause *reclarit* at any time and are reminded to take breaks. The program sends regular tailored messages via email or SMS to foster sustained engagement with therapeutic content and exercises. *reclarit* can be used for a period of 180 days.

Participants in the control group received educational material on lifestyle in RA provided by the German Rheumatism league in PDF format via email. Following the pragmatic study design, TAU was not specified but allowed to reflect the reality of routine care and therefore comprised all forms of care, including medication, psychotherapy, or no treatment at all.

### Data security

Study employees were trained to treat all information confidentially. Participants’ names and contact information were known only to the study manager. Personal email addresses and other personal data were removed from the data record. Research data were pseudonymized during the study period and stored in encrypted folders on secure servers.

### Outcomes and assessments

The primary endpoint was health-related QoL, as assessed by the SF-36 (RAND version of the Medical Outcomes Study)^34^, at 3 months in the ITT population. The SF-36 is a validated self-report questionnaire consisting of 8 scales with 36 questions. Responses are used to derive two component scores, the MCS and PCS, on a scale of 0 to 100, with lower values indicating greater impairment. This instrument is frequently used in clinical trials of RA^58,59^.

Secondary endpoints included the SF-36 at 6 months and assessments of other PROs at 3 and 6 months. Depression was assessed with the PHQ-9, which ranges from a score of 0 (no depression) to 27 (severe depression). Anxiety was assessed by the GAD-7 questionnaire with scores ranging from 0 (none) to 21 (severe). Fatigue was assessed by the Bristol RA Fatigue Multi-Dimensional Questionnaire (BRAF-MDQ), which consists of 20 questions about symptoms over the past 7 days and is scored on a scale of 0 (none) to 70 (severe). Social and work functioning was assessed by the Work and Social Adjustment Scale (WSAS); lower scores correspond to less impairment. Pain was assessed by a numeric rating scale (NRS) ranging from 0 (no pain) to 10 (worst possible pain). Physical function was assessed by the HAQ-DI; lower scores correspond to less impairment. References for these instruments can be found in Supplementary Section S1.

User satisfaction was assessed in a post-hoc analysis with the NPS in which participants were asked how likely they were to recommend *reclarit* to a friend or colleague on an 11-point NRS ranging from 0 (would definitely not recommend) to 10 (would definitely recommend). The percentage of respondents who selected ratings of 0 to 6 (detractors) was subtracted from the percentage who selected 9 or 10 (promoters) to provide an NPS^60^.

### Statistical analysis

The required sample size for the study was calculated by use of an a priori power analysis using G*Power (version 3.2.9.4) and based on previous studies on psychosocial outcomes in RA^20,36,41^. These calculations determined that 352 participants were required to achieve a power of 0.8 for the primary outcome. The calculated sample size was not corrected for expected attrition because missing values were replaced by multiple imputation (see below).

The primary analysis of SF-36 at 3 months was performed on the ITT population with multiple imputation using a “missing at random” assumption. Missing data points were imputed using variable values at baseline and other specified sociodemographic and clinical variables (see Supplementary Section S2 for additional details). ANCOVA analyses were performed with the analyzed outcome at 3 months serving as the dependent variable and baseline values of the outcome as the covariate. Treatment effects (baseline-adjusted mean group differences between the *reclarit* and control groups) were reported along with the corresponding 95% confidence interval (CI). The two-sided p value of the treatment effect from the ANCOVA was used to determine statistical significance of the results. A Bonferroni correction was applied to SF-36 MCS and PCS to account for multiplicity and control for family-specific error rate; for these analyses, p < 0.025 was considered statistically significant. For all other evaluations, p < 0.05 was considered statistically significant. Between-group effect sizes (Cohen’s *d*)^61^ were determined based on the difference in observed mean values between the *reclarit* and control groups at 3 months. These statistical methods were also used to evaluate outcomes at 6 months and in PP analyses at 3 and 6 months.

Analyses of therapy use in the *reclarit* vs control groups at 3 and 6 months were performed with the chi-square goodness-of-fit test.

A post-hoc responder analysis was performed on the SF-36 MCS outcome in the ITT population at 3 months using the reliable change index (RCI) and a *z*-score cut-off of 1.96^62^ to define responders as participants who showed reliable improvements from baseline to the 3-month time point. Responder analyses were also performed to evaluate changes in depression (using a minimum clinically important difference change of 5 points in the PHQ-9 total score^63^) and changes in anxiety (using a change of 4 points in the GAD-7 score^64^) in the ITT population. Post-hoc subgroup analyses were used to evaluate the potential impact of demographic and clinical characteristics on PROs.

All analyses were performed with *R*, version 4.2.1.

## Supplementary information

Supplementary Information

## Data Availability

The datasets used and/or analyzed during the current study are not publicly available due to proprietary reasons but are available from the corresponding author on reasonable request.
