# Supplementary material for: Efficacy of a cognitive-behavioral digital therapeutic on psychosocial outcomes in rheumatoid arthritis: randomized controlled trial
Source: Npj Ment Health Res. 2024 Sep 3;3:41. doi: 10.1038/s44184-024-00085-8 (PMC11371912; doi:10.1038/s44184-024-00085-8)
Supplement: Supplementary file 1 — Supplementary Information [file 44184_2024_85_MOESM1_ESM.pdf]

Supplementary Information

Efficacy of a Cognitive-Behavioral Digital Therapeutic on Psychosocial Outcomes in Rheumatoid Arthritis: Randomized Controlled Trial

Linda T. Betz, Gitta A. Jacob, Johannes Knitza, Michaela Koehm, Frank Behrens

Supplementary Section S1. Instruments used to assess secondary endpoints

Outcome	Instrument	Scoring range	Reference
Depression	Patient Health Questionaire-9	0 (no depression) to 27 (severe depression)	1
Anxiety	Generalized Anxiety Disorder-7	0 (none) to 21 (severe)	2
Fatigue	Bristol Rhematoid Arthritis Fatigue Multi-Dimensional Questionnaire	0 (none) to 70 (severe)	3
Social and work functioning	Work and Social Adjustment Scale	0 (no impairment) to 40 (severe impairment)	4
Pain	Numeric rating scale	0 (no pain) to 10 (worst possible pain)	5
Physical function	Health Assessment Questionnaire-Disability Index	0 (no disability) to 3 (completely disabled)	6

Supplemental Section S2. Statistical analyses

In analyses of outcomes, missing data points were imputed using the respective variable values at baseline as well as group membership and other sociodemographic and clinical variables (age, sex, intake of anti-rheumatic medication at baseline, physiotherapy at baseline, psychotherapy at baseline). Intention-to treat (ITT) and per-protocol analyses were implemented following a computationally efficient implementation for bootstrapped maximum likelihood multiple imputation by von Hippel and Bartlett[1] using the *R* packages *bootImpute* and *mice*.[7,8] A total of 1,000 bootstrap samples of the incomplete data set (with the above-mentioned variables) were generated for each outcome variable and then the relevant outcome variable was imputed twice using the *mice* package with default settings (i.e., using the predictive mean matching method with a pool of 5 candidate values drawn at random).

For responder analyses for the Short Form-36 mental component summary (SF-36 MCS) in the ITT population, we used the reliable change index (RCI) to define responders as participants who showed reliable improvements from baseline to the 3-month time point in the SF-36 MCS. Specifically, we considered the change to be clinically relevant if the RCI value exceeded a critical *z*-score of 1.96 for a 95% confidence interval for a given participant. In each of the 2,000 imputed data sets, the imputed outcomes were dichotomized into responders and non-responders based on RCI, and then the proportions of responders in the *reclarit* and control groups were compared.

References

1 Kroenke, K., Spitzer, R.L. & Williams, J.B.. The PHQ-9: validity of a brief depression severity measure. *J. Gen. Intern. Med.* **16**, 606-13 (2001). doi: 10.1046/j.1525-1497.2001.016009606.x.

2 Spitzer, R.L., Kroenke, K., Williams, J.B. & Löwe, B. A brief measure for assessing generalized anxiety disorder: the GAD-7. *Arch. Intern. Med.* **166**, 1092-7 (2006). doi: 10.1001/archinte.166.10.1092.

3 Nicklin, J. et al. Measuring fatigue in rheumatoid arthritis: a cross-sectional study to evaluate the Bristol Rheumatoid Arthritis Fatigue Multi-Dimensional questionnaire, visual analog scales, and numerical rating scales. *Arthritis Care Res. (Hoboken)* **62**, 1559-68 (2010). doi: 10.1002/acr.20282.

4 Heissel, A. et al. Validation of the German version of the work and social adjustment scale in a sample of depressed patients. *BMC Health Serv. Res.* **21**, 593 (2021). doi: 10.1186/s12913-021-06622-x.

5 Williamson, A. & Hoggart, B. Pain: a review of three commonly used pain rating scales. *J. Clin. Nurs.* **14**, 798-804 (2005). doi: 10.1111/j.1365-2702.2005.01121.x.

6 Bruce, B. & Fries, J.F. The Health Assessment Questionnaire (HAQ). *Clin. Exp. Rheumatol.* **23(5 Suppl 39)**, S14-8 (2005).

7 von Hippel, P.T. & Bartlett, J.W. Maximum likelihood multiple imputation: faster imputations and consistent standard errors without posterior draws. *Statistical Science* **36**, 400-20 (2021). doi: 10.1214/20-STS793.

8 van Buuren, S. & Groothuis-Oudshoorn, K. mice: multivariate imputation by chained equations in R. *Journal of Statistical Software* **45**, 1-67 (2011). doi: 10.18637/jss.v045.i03.
